# Oral Tolerance to Cancer Can Be Abrogated by T Regulatory Cell Inhibition

**DOI:** 10.1371/journal.pone.0097602

**Published:** 2014-05-15

**Authors:** Maria C. Whelan, Garrett Casey, John O. Larkin, Barbara-ann Guinn, Gerald C. O'Sullivan, Mark Tangney

**Affiliations:** 1 Cork Cancer Research Centre, BioSciences Institute, University College Cork, Cork, Ireland; 2 Department of Life Sciences, University of Bedfordshire, Park Square, Luton, United Kingdom; Centro di Riferimento Oncologico, IRCCS National Cancer Institute, Italy

## Abstract

Oral administration of tumour cells induces an immune hypo-responsiveness known as oral tolerance. We have previously shown that oral tolerance to a cancer is tumour antigen specific, non-cross-reactive and confers a tumour growth advantage. We investigated the utilisation of regulatory T cell (Treg) depletion on oral tolerance to a cancer and its ability to control tumour growth. Balb/C mice were gavage fed homogenised tumour tissue – JBS fibrosarcoma (to induce oral tolerance to a cancer), or PBS as control. Growth of subcutaneous JBS tumours were measured; splenic tissue excised and flow cytometry used to quantify and compare systemic Tregs and T effector (Teff) cell populations. Prior to and/or following tumour feeding, mice were intraperitoneally administered anti-CD25, to inactivate systemic Tregs, or given isotype antibody as a control. Mice which were orally tolerised prior to subcutaneous tumour induction, displayed significantly higher systemic Treg levels (14% vs 6%) and faster tumour growth rates than controls (p<0.05). Complete regression of tumours were only seen after Treg inactivation and occurred in all groups - this was not inhibited by tumour feeding. The cure rates for Treg inactivation were 60% during tolerisation, 75% during tumour growth and 100% during inactivation for both tolerisation and tumour growth. Depletion of Tregs gave rise to an increased number of Teff cells. Treg depletion post-tolerisation and post-tumour induction led to the complete regression of all tumours on tumour bearing mice. Oral administration of tumour tissue, confers a tumour growth advantage and is accompanied by an increase in systemic Treg levels. The administration of anti-CD25 Ab decreased Treg numbers and caused an increase in Teffs. Most notably Treg cell inhibition overcame established oral tolerance with consequent tumor regression, especially relevant to foregut cancers where oral tolerance is likely to be induced by the shedding of tumour tissue into the gut.

## Introduction

Even allowing for comparable tumour stages the prognosis for patients suffering from oesophageal and gastric cancer remains consistently and significantly poorer than for patients with distal gastrointestinal tract cancers, despite advances in diagnostic, surgical and adjuvant therapies [1], [2]. Among the many variables that determine tumour growth rates and prognoses, differences in tumour immune responsiveness are likely to exist between foregut and other cancers. The processing of dietary antigens (Ags) by the mucosal immune system in the gastro-intestinal tract leads to a systemic Ag specific immune hypo-responsiveness termed oral tolerance [3]. It is likely that tumour Ags derived from tumour tissue shed into the intestine by foregut cancers would be processed by the gut associated lymphoid tissues (GALT), predominantly found in the proximal gastrointestinal tract, in a way reminiscent of Ags ingested by the mucosal immune system, thus creating a tumour Ag specific immune tolerance. We previously reported that orally administered fresh tumour tissue induced a tumour Ag specific non-cross-reactive immune tolerance with a consequent growth advantage for the cancer [4].

The mechanism of tolerance to ingested Ags may be attributed to either active suppression or the induction of clonal deletion/anergy [5]. T cells cloned from tolerised mice have been ascribed to a unique subset of the CD4^+^ population, the Th3 cell [6]. In T cell receptor (TCR) transgenic mice, there was an increase in CD4^+^CD25^+^ cells in response to oral Ag administration. These Tregs were found to express CTLA-4 and foxp3 and to have a suppressive function *in vitro*. CD4^+^CD25^+^foxp3^+^ Tregs play a role in preventing the development of autoimmune disease and have a dual property as both anergic and suppressive cells. Significantly, it has been shown that removal of this population may induce anti-tumour immune activity [7]–[10]. In experimental systems, as tumours grow, there is a numerical increase in Tregs in the immune infiltrate with consequent local suppression of the anti-tumour immune responses. Antibody (Ab) mediated removal of these Tregs can unmasks natural tumour immune reactivity and this strategy has been shown to potentiate the immunotherapeutic destruction of experimental cancers [10]–[12].

We have investigated the effects of oral tolerance to the JBS tumour on tumour growth rates and on the size of lymphocyte subpopulations in the Balb/C mouse. Specifically we examined if the induction of oral tolerance to tumours was Treg-dependent – and whether Treg inactivation could abrogate oral tolerance and inhibit tumour growth.

## Materials and Methods

### Cell Tissue Culture

The JBS murine fibrosarcoma [13] was grown in tissue culture flasks at 37°C in a humidified atmosphere of 5% CO_2_, in DMEM (Dulbecco's Minimal Essential Medium –Sigma-Aldrich, Dublin, Ireland). The tissue culture media were supplemented with 10% iron-supplemented donor calf serum, 50 µg/ml gentamycin, 300 µg/ml L-glutamine, and 10 mM HEPES (1-Piperazineethane sulfonic acid, 4-(2-hydroxyethyl) monosodium salt), pH 7.4. Standard procedures for trypsinisation, centrifugation and resuspension of cells were used [13]. Viable cell counts were conducted by using Trypan Blue Dye Exclusion (Sigma, Ireland).

### Ethics Statement

All murine experiments were approved by the animal ethics committee of University College Cork (AERR #2010/003). The study was carried out in strict accordance with the recommendations laid down by the Irish Department of Children and Health.

### Tumour Induction

Mice were obtained from Harlan Laboratories (Oxfordshire, England). They were kept at a constant room temperature (22°C) with a natural day/night light cycle in a conventional animal colony. Standard laboratory food and water were provided *ad libitum*. Before experiments, the mice were afforded an adaptation period of 14 days. Balb/C mice of both sexes and athymic male Balb/C HsdOla:MF1-nu mice in good condition, weighing 16–22 g at 6–8 weeks of age, were included in experiments. For JBS tumour induction, 2×10^6^ tumour cells, suspended in 200 µl DMEM, were injected subcutaneously into the flank of the mice. With this volume of inoculum and location there was little puncture site extravasation of the cell suspension and the tumour growth and shape was consistent.

### Gavage Feeding

Following subcutaneous inoculation of 2×10^6^ JBS tumour cells in Balb/C mice, tumours developed and were allowed to attain a size of 1–2 cm^3^, at which stage the animals were sacrificed and their tumours were removed. The tumours were homogenized in phosphate buffered saline (PBS) (Sigma, Ireland) using a FastPrep FP120 homogeniser (Thermo Electron Corp., England). The homogenate was extensively washed by centrifugation with PBS to remove debris. When the supernatant was macroscopically clear the homogenate was resuspended in PBS to a final concentration of 0.2 g (wet weight) per ml. This was aliquoted and frozen until use. The PBS control was also aliquoted and frozen. Using an 18 Fr-steel-ball-tipped feeding needle attached to a 1 ml syringe, un-anaesthetised animals were gavage fed 200 µl of freshly thawed JBS tumour homogenate (40 mg of tumour) or PBS. Animals were fed daily for 14 days. The amount of feed and the duration of feeding corresponded to those used in previously published studies by our group and were based on a literature review, particularly in regard to studies that examined tolerance to cellular Ags [4], [14]–[17]. On day 15, animals received a subcutaneous inoculation of tumour cells (2×10^6^ cells) and were monitored for tumour development daily.

### Tumour Monitoring

Tumours were measured every 48 hours using a digital calliper. Tumour volume was calculated using the standard formula *v* = *ab* ^2^π/6 and tumour growth curves were constructed. The mice were humanely euthanised in instances where the tumour reached 1.5 cm^3^ in diameter. In the first experiment, mice were fed tumour Ag or PBS, and growth and survival curves were prepared. In the second experiment, systemic Tregs were either permanently inactivated (referred to as depleted) using anti-CD25 Ab (clone PC61- Bio-Express, New Hampshire, USA), temporarily depleted during feeding only or not depleted. In the final experiment mice were fed for 14 days and subsequently randomised to receive anti-CD25 Ab or a control Ab conjugated to anti-horse radish peroxidase (HPRN), Bio-Express, USA to monitor the effect on sc tumour growth.

### Flow Cytometry Analysis

ACK erythrocyte lysing buffer (0.15 M NH_4_Cl, 10 mM KHCO_3_, 0.1 mM Na_2_EDTA, pH 7.4 with HCl) was prepared using reagents purchased from Sigma-Aldrich, Ireland. The solution was filter sterilised through a 0.2 µm filter and stored at 4°C. FACS staining buffer was prepared from Dulbecco's phosphate-buffered saline (Dulbecco's PBS) purchased from Sigma along with, bovine serum albumin (BSA), sodium azide and foetal calf serum. Spleens and tumour tissue were removed and minced separately on 70 µm nylon cell strainer (BD Biosciences, UK). These cell suspensions were collected, washed in culture medium, pelleted at 300 g for 10 min at 4°C and were then treated with ACK erythrocyte lysing buffer for 3 min after which they were washed twice and resuspended in FACS staining buffer. Viable cell numbers were determined by a standard Trypan Blue Exclusion test. Cells were then diluted to a concentration of approximately 2–4×10^6^ per ml and 100 µl staining buffer added per Falcon tube (Becton Dickinson). Cell suspensions and reagents were maintained at 4°C during preparation.

### Cell Surface Marker Analysis

Phycoerythrin–Cy5 (PE-Cy5) conjugated antibodies against murine CD25 and CD3, fluorescein isothiocyanate (FITC)-conjugated antibodies against murine CD25, CD3, CD4 and CD8 and the PE anti-mouse foxp3 staining kit were purchased from eBioscience, Insight Biotechnology, England. PE anti murine CD4 and CD3 were purchased from Serotec, Oxford, England. Fluorochrome-conjugated isotype staining controls (FITC-IgG, PE-IgG2a and PE-Cy5 IgG Isotype Control) were purchased from eBioscience. Unconjugated anti-murine CD16/CD32 (Fcy III/II) monoclonal Ab (Fc receptor blocking Ab) was purchased from Serotec.

In order to minimise non-specific Ab binding, anti-CD16/CD32 Fc receptor blocking Ab was diluted to 0.01 µg/µl in staining buffer, 20 µl per well added and incubated at 4°C for 20 min. Fluorochrome-conjugated antibodies to cell surface markers were also diluted as per manufacturer's instructions and were added to each sample without removal of the Fc-blocking Ab. Cells were incubated in darkness at 4°C for 45 min then washed twice in 250 µl staining buffer before resuspension in 500 µl staining buffer for immediate analysis or a fixative solution of 0.5% paraformaldehyde in Dulbecco's PBS. Analysis was performed within 48 hours using a FACScaliber flow cytometer (Becton Dickinson, Oxford, United Kingdom) and analysed by the accompanying CELLQuest (BD Biosciences) computer software program.

### *In Vivo* Ab Administration

**Figure 1:**
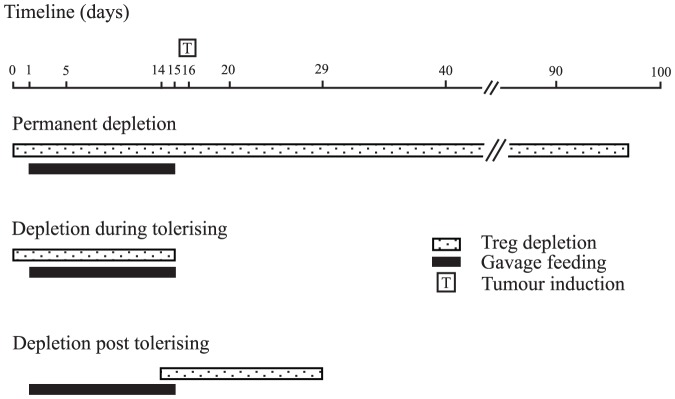
Schematic representation of experimental protocols. Initial experiments involved mice which were depleted of Tregs for the duration of experiment and were referred to as being permanently depleted. For experiments involving depletion during tolerisation, Treg depletion only occurred during tumour feeding and not when tumours were induced. The final protocol (depletion post tolerisation) required oral tolerance to be established prior to Treg depletion.

As previously stated, anti-CD25 Ab (PC61) and control Ab (isotype control rat IgG-HRPN) were administered intra-peritonealy at a dose of 1 mg/kg in a total volume of 200 ul of PBS. The timing of doses depended on the experimental protocol but when two doses were to be administered they were given four days apart (Fig. 1). This resulted in over 95% inactivation of Tregs as determined by flow cytometry.

### Statistical Analysis

The differences between the individual groups were tested using the two-tailed Student's *t*-test for paired values. Differences with a *p* value less than 0.05 were considered significant.

## Results

### Oral Administration of Tumour Tissue Confers a Tumour Specific Growth Advantage

**Figure 2:**
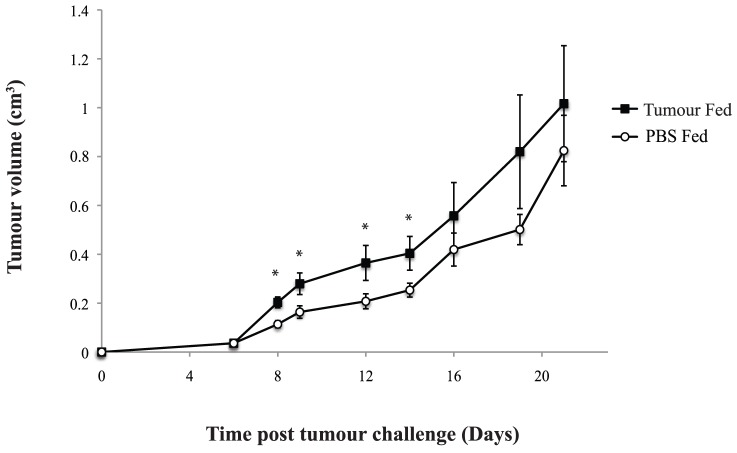
Subcutaneous tumour growth in immune-competent gavage fed mice. Growth curves for JBS tumours in mice following gavage feeding for 14 consecutive days with JBS tumour homogenate or with PBS. JBS tumours grew significantly faster in mice orally tolerised with JBS. Each point represents the mean tumour volume of a panel of six mice. The difference was statistically significant (p<0.05) at days 8, 9, 12 & 14 as indicated by an asterix on the graph.

We have previously shown that subcutaneous tumours have a faster growth rate in mice that were fed tumour prior to tumour induction, compared with mice that were fed either PBS or an alternative tumour (CarB or CT26) [4], [13]. We have also demonstrated that the tumour growth curve in Balb/C mice approximates the growth curve of subcutaneous tumour in athymic nude mice, which lack functioning T cells and these mice were used as an immune incompetent control [13]. In this study, using the same feeding protocol, we validated that our tumour feeding regime resulted in a consistent and significantly increased subcutaneous tumour growth rate in Balb/C mice versus control groups. Subcutaneous tumours in the groups fed JBS tumour appeared earlier (day 5 vs. day 6/7) and grew significantly faster (p<0.05) at several time points (Fig. 2).

### Flow cytometry analysis of lymphocytes in response to tumour ingestion

**Figure 3:**
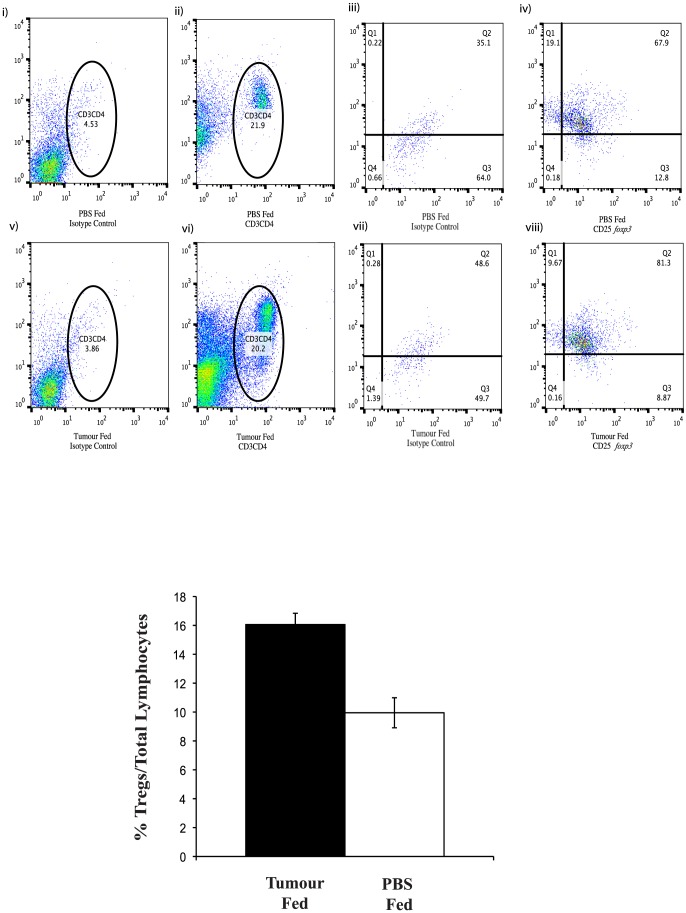
Peripheral Treg numbers in mice gavage fed tumour or PBS. (**a**) Dot plots show the number of peripheral blood Tregs in PBS (i–iv) or JBS (v-,viii) fed mice. Tregs were stained using CD3, CD4, CD25 and *foxp3* antibodies conjugated to fluorochromes and isotype controls and analysed on the flow cytometer. The cells were gated on CD3^+^ and CD4^+^ (ii and vi) and isotype controls (i and v) and subsequent dot plots were acquired through this gate for CD25^+^ and *foxp3* ^+^ cells (iv and viii) and isotype controls (iii and vii). There was a significant increase in CD4^+^CD25^+^ cells (p<0.022) following tumour administration versus PBS. Moreover there was a significant increase observed in CD25^+^/*foxp3* ^+^ expression following gating on the CD3^+^CD4^+^ population of lymphocytes (p<0.019). (**b**) Graph representing the mean percentage values of Treg numbers minus the isotype controls within the total peripheral lymphocyte population from JBS or PBS fed mice. There was significantly more Tregs in the spleens of mice that were fed homogenised tumour for 14 days than those fed PBS alone (p<0.002; n = 6).

Following 14 days of oral tumour or PBS administration, splenic lymphocyte populations were analysed. There was a significant increase in CD4^+^CD25^+^ cells (p<0.022) following tumour administration versus PBS. Moreover there was a significant increase observed in CD25^+^/*foxp3* ^+^ expression following gating on the CD3^+^CD4^+^ population of lymphocytes (p<0.019) (Fig. 3). There was no significant increase in total numbers of CD3^+^CD4^+^ or CD3^+^CD25^+^ cells. Neither was there any significant increase in the CD8^+^CD25^+^ Teff sub-group populations following the tumour feeding schedule. There was no observable difference in the number or ratio of lymphocyte populations between those mice which were fed PBS and those not gavage-fed during this period (data not shown).

### Increased Tumour Growth Rate is Associated with the Activity of Tregs

We have previously shown that our anti-CD25 Ab administration protocol facilitates 90% inactivation of CD25^+^ cells over a period of 14 days [8]. Mice were randomised to receive either anti-CD25 Ab at the commencement of the 14-day feeding schedule, and again at the end of feeding (referred to as permanent depletion) or Ab at the time of commencing feeding only (referred to as depletion during tolerising) or control Ab, or no Ab (Fig. 1).

**Figure 4:**
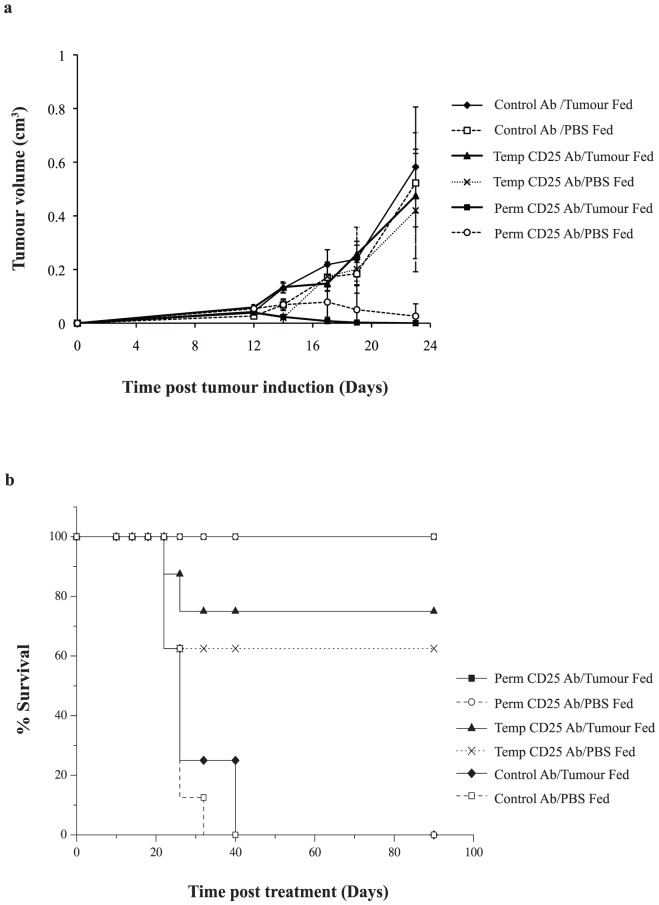
Tumour growth and survival following anti-CD25 Ab. (**a**) There was no significant difference between tumour growth rates between mice fed tumour (temporarily or permanently) and control mice. Mice which received no anti-CD25 Ab depletion showed significantly different tumour growth rates at days 14, 18 and 22 depending on whether they had been fed tumour and saline (p<0.05). Each data point represents mean tumour values for 10 mice. (**b**) Survival curve for mice that were permanently, temporarily or not depleted during the induction of oral tolerance. Mice that were permanently depleted were 100% cured of their subcutaneous tumour and remained disease free at 100 days. 75% of those that were temporarily depleted while fed tumour were cured, while 66% of those that were temporarily depleted and fed PBS cured. There was no significant difference between these groups. All of those mice that received isotype control Ab succumbed to tumour burden (n = 10).

The resulting subcutaneous tumour growth curves showed that the previously observed tumour growth advantage following feeding was absent in mice undergoing the depletion during the tolerising schedule, with time to tumour appearance and tumour growth rate similar to PBS-fed mice, indicating the requirement for Tregs at the time of tumour feeding for the induction of oral tolerance (Fig. 4a). As seen in Fig. 4b, tumours in mice receiving anti-CD25 Ab eventually regressed completely, both in depletion during tolerisation and permanent depletion groups. We, and others, have previously reported the finding that anti-CD25 administration prior to tumour induction induces complete tumour regression [8].

### Changes in Lymphocyte Sub-populations Observed in Response to the Depletion of Tregs Either Permanently or During Tolerisation

**Figure 5:**
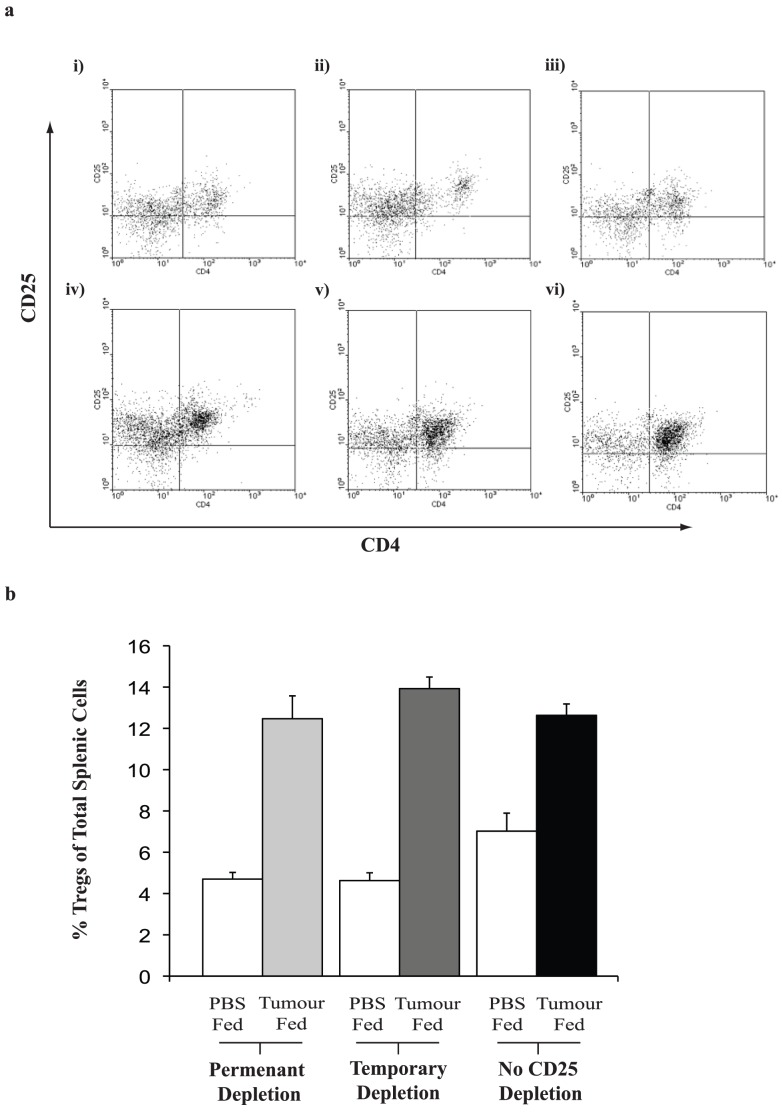
Splenic Tregs following feeding and Ab therapy. (**a**) Cells were stained for CD3, CD4 and CD25. The cells were initially gated on CD3 and subsequently on CD4^+^CD25^+^. Splenic Tregs were analysed from mice which had been treated with (i) isotype control antibody followed by PBS feeding; (ii) temporarily depleted with anti-CD25 antibody but PBS fed (iii) permanently depleted with anti-CD25 antibody and PBS fed; (iv) isotype control antibody treatment and tumour fed, (v) temporarily depleted with anti-CD25 but tumour fed or (vi) permanently depleted and tumour fed. Representative dot plots from relevant groups are shown, three mice from each treatment group were analysed; (**b**) Graph representing the mean data from the dot plots in (a). There is a significant difference between those mice that were tumour fed or PBS fed and permanently depleted with anti-CD25 (p<0.01), depletion during tolerisation while PBS or JBS fed (p<0.001), control Ab, tumour fed versus control Ab, PBS fed (p<0.01). There was a significant difference between those mice that were treated with control Ab or PBS fed and those that were treated with anti-CD25 Ab at any time and PBS fed, (p<0.05). There was no significant difference in Tregs between any of those mice that were tumour fed across the treatment groups.

At various time points, mice from each group were sacrificed and their spleens analysed for systemic CD4^+^CD25^+^ Treg and CD8^+^CD25^+^ Teff numbers. Tumour feeding significantly increased systemic Treg numbers versus PBS fed groups, and this difference was also seen in anti-CD25 Ab administered mice (Fig. 5a) (p<0.01). While there were significantly lower numbers of Tregs in PBS-fed groups that received anti-CD25 Ab than those who received control Ab (Fig. 5b) (p<0.05), surprisingly, a reduction in overall Treg numbers was not found in anti-CD25 Ab, tumour-fed mice.

**Figure 6:**
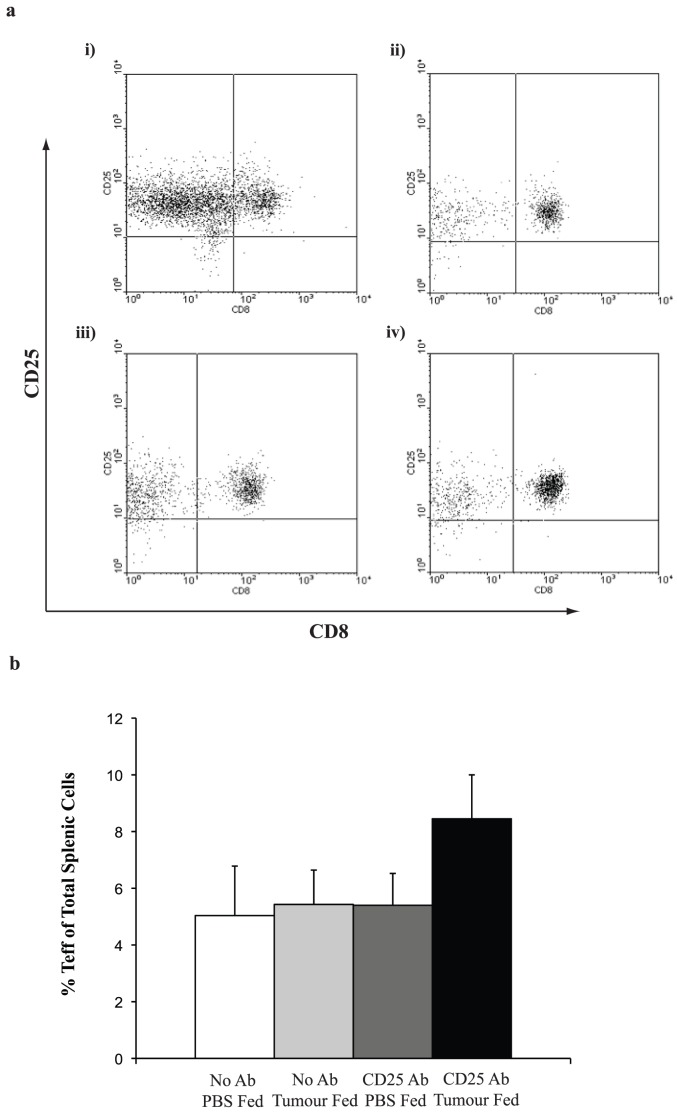
Splenic Teff cells following feeding and Ab therapy. (**a**) Spleens from mice in each treatment group were stained to determine CD3^+^/CD8^+^ and CD3^+^/CD25^+^ numbers. Groups were as follows (i) tumour fed but no antibody depletion; (ii) PBS fed and no antibody depletion; (iii) PBS fed and anti-CD25 treated and (iv) tumour fed, anti-CD25 treated. CD8^+^ and CD25^+^ were examined from within the CD3^+^ population. Each experiment was performed in triplicate; (**b**) Graph representing the changes in Teff numbers following tumour feeding and Ab depletions. Cumulative data from dot plots represented in (**a**) (n = 3).

We also examined the effect of oral tolerance and anti-CD25 Ab on the systemic activated effector lymphocyte population, namely the CD8^+^CD25^+^ cells. There was an increase in the number of Teff cells that were permanently depleted and fed tumour compared with all other groups (Fig. 6) and this value was approaching significance.

### Anti-CD25 Ab Therapy Post-tumour Feeding Overcomes the Induced Oral Tolerance

**Figure 7:**
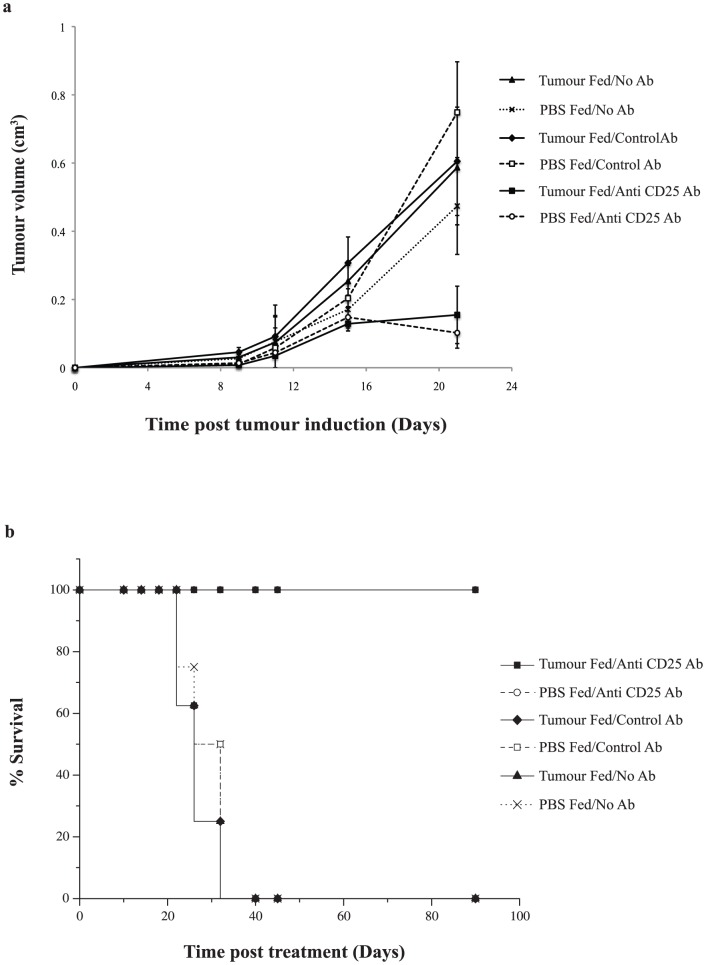
Anti-CD25 Treg depletion therapy overcomes established oral tolerance. (**a**) Mice were fed tumour or PBS and randomly assigned to receive anti-CD25 Ab, isotype control Ab or PBS. Those which received anti-CD25 Ab had slower tumour growth rate compared to the other groups. The significant difference in growth rate between those that were tumour or PBS fed was maintained in the isotype control Ab and no Ab groups at certain time points (p<0.05); (**b**) Mice that were tolerised to tumour antigen through oral administration of tumour were subsequently treated with anti-CD25 Treg depletion treatment and were cured of their subcutaneous tumour remaining tumour free for at least 100 days.

To determine whether anti-CD25 Ab therapy can abrogate an established tumour growth advantage, mice which had completed the oral tumour feeding schedule were randomly divided into groups to receive anti-CD25 Ab versus control Ab or no Ab (PBS alone). Administration of anti-CD25 Ab immediately following tumour feeding, removed the previously observed tumour growth advantage (Fig. 7a). This was found to be significant when compared with the other groups (p<0.03). Survival data showed that 100% of mice which received anti-CD25 Ab were cured, a cure rate equivalent to the group of mice not fed tumour and subsequently treated with anti-CD25 Ab (Fig. 7b).

### Treg Numbers in Cured Mice

**Figure 8:**
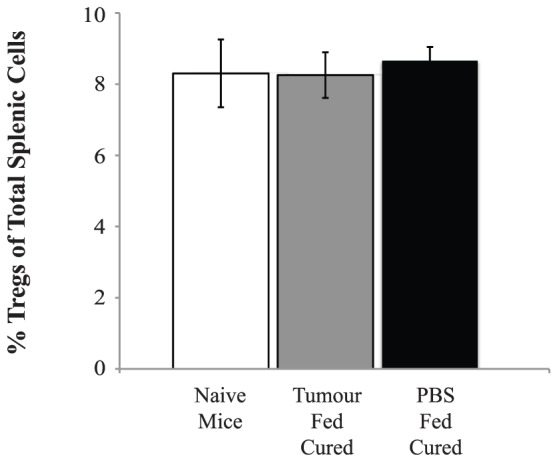
Tregs in cured mice. One hundred and thirty days after being treatment with anti-CD25 antibody, mice were sacrificed and their spleens stained for CD4^+^CD25^+^ Tregs. There were no significant differences seen in the percentage of CD4^+^CD25^+^ cells between those mice that had cured of their tumour or naïve mice (n = 3 per group).

The anti-CD25 Ab treatment did not have long term effects on Treg numbers. Mice cured of their tumours through anti-CD25 administration were studied 30 days later and had similar Treg numbers to naïve animals irrespective of whether they were from the orally tolerised or control groups (Fig. 8).

## Discussion

Most patients who develop cancers of the upper gastrointestinal tract die as a direct consequence of their disease. Even for those with clinically localized tumours, who are subjected to curative surgery, the five year relapse rates are usually above sixty percent [18]. At the time of surgery, most of these patients have micrometastases in various tissues indicating haematogenous spread of the cancer at an early stage of tumour development. This leaves patients in a minimal disease state post-surgical treatment. A therapy that could facilitate the immune system to identify tumour Ags systemically would be ideal, if not to clear all of the disease burden, then at least to create a state of tumour dormancy [19],[20].

We have previously shown that growth of the weakly immunogenic JBS cell line in the flank of immune competent mice led to an increase in Treg cell numbers in the tumour environment but not systemically [8]. In this study we have modelled the presence of tumour fragments in the gut to the clinical situation by gavage feeding of JBS cells to mice. We would expect these fragments to be processed by the GALT and would result in a systemic hypo-responsiveness to tumour Ags [13]. We have demonstrated for the first time that CD4^+^CD25^+^ Tregs are systemically increased in response to oral administration of whole (JBS) tumour. In addition we have shown that the induction of oral tolerance to a tumour confers a growth advantage to the cancer.

Mice whose Tregs were depleted by anti–CD25 treatment, irrespective of tumour or PBS feeding, demonstrated regression of tumours and long term cures. There were no differences in response rates between the tumour fed and control groups indicating that Treg inhibition effectively overcomes the combined oral tolerance and tumour growth influences. It is likely that the early induction of Tregs at the systemic level, achieved in this study by tumour feeding, is responsible for the tumour growth advantage observed. It is of interest that there were no differences in the numbers of CD8^+^ T cells between any of the groups suggesting that a reduction in Teffs does not occur with oral tolerance induction. It is also likely that oral tolerance and early Treg responses did not inhibit immune sensitisation to the tumour but inhibited effector function as there were no differences in the timing or completeness of tumour regression after Ab treatment between the tumour fed tolerised or the PBS control group of mice. There was a significant difference in the total number of CD4^+^CD25^+^ Tregs in mice which received anti-CD25 Ab and were tumour fed and the control saline fed groups. This was also observed in those groups which were temporarily depleted. Surprisingly however, there was no significant decrease in Tregs observed between the groups that were tumour fed and received either a dosing schedule of anti-CD25 Ab or isotype control Ab. A possible explanation for this may be due to the analysis of Treg numbers using CD4^+^CD25^+^ only at this stage of the experimental process as opposed to the more specific CD4^+^CD25^+^ *foxp3* ^+^. No significant decrease in Tregs may in fact involve the experimental protocol and the timing for examination of Tregs and dose of anti-CD25Ab, which was chosen based on our previous knowledge that maximum Treg depletion was within 7 days of Ab administration. The dose of Ab used in this study ensured a 90% reduction in Treg numbers in naïve mice. The overall increase in Treg numbers in response to feeding was not taken into consideration with an increased dose of Ab. Despite this, functional differences between different doses of anti-CD25 Ab were observed in the resulting growth curves, suggesting CD4^+^CD25^+^ Treg cells are involved in the T cell response that confers the tumour growth advantage following feeding. This may be due to the fact that the effect of removing or functionally inactivating Tregs with Ab depletions during the development of oral tolerance is enough to allow for partial education of the immune system and the development of anti-tumour cytotoxic responses. Interestingly, there were more cures seen in the groups that were given anti-CD25 Ab at the time of feeding only and fed tumour (75%) than in those given Ab at the time of feeding and fed PBS (60%). It may be that Ag loading with feeding and subsequent inactivation of Tregs giving rise to increased Teff numbers could have occurred and similar findings have been demonstrated elsewhere [21].

In the clinic, patients with GALT present with an already orally tolerised immune system, and it remains to be determined whether oral tolerance can be overcome after tumour growth has been established. In studies of tolerance, it has been shown that the adoptive transfer of Tregs into murine models can overcome immune mediated disease [5]. In this study, we examined whether the removal of Tregs can allow the development of a cytotoxic T cell response to a subcutaneous tumour. Following the feeding of tumour and establishment of oral tolerance, a single dosing schedule of anti-CD25 Ab removed the tumour growth advantage that would normally be seen in the absence of anti-CD25 Ab treatment. Moreover, the administration of anti-CD25 Ab led to cures in all mice harbouring subcutaneous tumours. We have shown that even an established oral tolerance to a tumour Ag, and the resulting aggressive tumour growth and poorer survival, can be overcome by Treg attenuating therapy. This result suggests that cancers of the upper gastrointestinal tract would be amenable to therapies that attempt to inactivate established Treg cell activity. It is also important to note that anti-CD25 Ab therapy did not affect the systemic Treg populations in treated mice following therapy, compared with naïve mice. This is important as it limits the possibility that anti-CD25 Ab therapy of autoimmune diseases could remove Tregs entirely or permanently.

The results of this study suggest a mechanism of tolerance – a T-cell-mediated suppression of an antitumour immune response. The identity of the tumour Ag(s) has yet to be established; however, this study confirms and expands on observations of previous reports where feeding of tumour Ags was shown to impair antitumour cytotoxicity and thus supports the hypothesis that tumour Ags which are shed into the upper gastrointestinal tract and processed by the mucosal immune system may result in down regulation of systemic antitumour immune responses [3], [22].

We have shown that processing of the shed tumour results in the generation of immunosuppressive Treg cell population which can attenuate the antitumour immune response and that the development of oral tolerance in this clinically based model is Treg-dependent. Moreover, we demonstrate that the abrogation of Treg cell function can be achieved with the use of anti-CD25 Ab giving rise to a systemic, non-toxic, effective anti-tumour response. Our studies show that immune modulatory effects may contribute to the significantly poorer prognosis in foregut cancers and may be overcome by immune therapy, representing a significant potential advance in the development of more effective therapies against these diseases. Anti-CD25 Treg depletion therapy has the capacity to improve prognosis and confer a survival advantage on patients with oesophageal and gastric cancers.
